# REGAIN: a randomized controlled clinical trial of oxaloacetate for improving the symptoms of long COVID

**DOI:** 10.3389/fnins.2025.1627462

**Published:** 2025-07-18

**Authors:** Suzanne D. Vernon, Candace Rond, Jennifer Bell, Brindisi Butler, Sara Isolampi, Annaleah Otteson, Pearl Phalwane, Samantha Mower, Shad Roundy, David L. Kaufman, Alan B. Cash, Lucinda Bateman

**Affiliations:** ^1^Bateman Horne Center, Salt Lake City, UT, United States; ^2^Department of Mechanical Engineering, University of Utah, Salt Lake City, UT, United States; ^3^Center for Complex Diseases, Seattle, WA, United States; ^4^Terra Biological LLC, San Diego, CA, United States

**Keywords:** oxaloacetate, long COVID, fatigue, cognitive impaiment, randomized clinical trial

## Abstract

**Background:**

Long COVID is characterized by fatigue, cognitive dysfunction, and other persistent symptoms. This randomized, double-blind, controlled trial evaluated the efficacy of oral oxaloacetate (OAA) in improving fatigue and cognitive function in adults with long COVID.

**Methods:**

A total of 69 participants were randomized to receive either 2,000 mg/day of OAA or the control for 42 days. The primary outcome was fatigue reduction, measured by the Chalder Fatigue Questionnaire (CFQ). The secondary and exploratory outcomes included the DePaul Symptom Questionnaire Short Form (DSQ-SF), health-related quality of life (RAND-36), cognitive function (Defense Automated Neurobehavioral Assessment (DANA) Brain Vital), and time upright (UP Time).

**Results:**

No significant difference in the CFQ-measured fatigue reduction was observed between the groups. However, the OAA group showed significantly greater improvements in the DSQ-SF-measured fatigue and total symptom burden at day 21 of the trial. Cognitive performance improved significantly in the OAA group, with strong correlations between symptom response and cognitive gains. OAA was well tolerated.

**Conclusion:**

OAA may contribute to earlier improvements in symptom burden and cognitive function in individuals with long COVID. Further studies are warranted.

## Introduction

Long COVID is an infection-associated chronic condition in which individuals continue to experience symptoms for months or years after the acute phase of COVID-19 has resolved (Committee on Examining the Working Definition for Long COVID, Board on Health Sciences Policy, Board on Global Health, Health and Medicine Division, and National Academies of Sciences, Engineering, and Medicine, 2024). These symptoms commonly include fatigue, cognitive impairment, shortness of breath, post-exertional malaise (PEM), and autonomic disturbances, although the clinical presentation and severity of illness can vary widely. Long COVID can affect anyone who has been infected with the SARS-CoV-2 virus, regardless of age, sex, or the severity of their initial illness (Sk Abd Razak et al., 2024). It is estimated that at least 6% of the more than 700 million confirmed COVID-19 cases reported globally have developed Long COVID (Hanson et al., 2022). This persistent and often disabling condition has placed a significant burden on public health systems, economies, and individual well-being, with no widely accepted treatment currently available (Board on Health Care Services, Health and Medicine Division and National Academies of Sciences, Engineering, and Medicine, 2024). As such, long COVID represents a pressing global health challenge requiring urgent research and therapeutic innovation.

Emerging research has implicated mitochondrial dysfunction (Szögi et al., 2025), altered redox homeostasis (Vlaming-van Eijk et al., 2024), and altered metabolism (Saito et al., 2024) as potential contributors to the persistence of symptoms in long COVID patients. Oxaloacetate, a key intermediate of the citric acid cycle, has demonstrated the potential to modulate cellular metabolism, enhance mitochondrial biogenesis, and reduce neuroinflammation in preclinical models (Wilkins et al., 2014). Recent clinical trials of oxaloacetate supplementation have shown promising effects in reducing fatigue in both myalgic encephalomyelitis/chronic fatigue syndrome (ME/CFS) and long COVID (Cash and Kaufman, 2022; Cash et al., 2024).

This article presents the results of REGAIN, a randomized, controlled clinical trial evaluating the safety and efficacy of oral oxaloacetate supplementation in long COVID patients. Our primary objective was to determine the safety and effectiveness of oxaloacetate in reducing fatigue. The effects of oxaloacetate supplementation on physical and cognitive impairment were also explored. These findings offer new insights into a possible therapeutic avenue for this debilitating condition.

## Methods

### Trial design

This was a single-center, randomized, double-blind, controlled clinical trial investigating the use of anhydrous enol-oxaloacetate (oxaloacetate, OAA) to reduce fatigue in long COVID patients. This trial used simple randomization with concealed allocation. Investigational product bottles were pre-labeled at a central site to blind site staff to their contents. At Visit 1, site staff dispensed the next-numbered product bottles to participants without knowledge of their contents and remained blinded until study closure. There were three in-person visits over the course of the 42-day trial: Visit 1 (Day 1), Visit 2 (Day 21), and Visit 3 (Day 42). The trial was conducted at the Bateman Horne Center (BHC), Salt Lake City, Utah, in accordance with good clinical practice and the Declaration of Helsinki and was approved by the Institute of Regenerative and Cellular Medicine Institutional Review Board (IRCM-2022-318). There were no changes to the trial design or methods once enrollment commenced. This trial was registered at ClinicalTrials.gov (NCT05840237) on 28 April 2023, https://clinicaltrials.gov/study/NCT05840237. Recruitment began in May 2023, and the trial was completed in December 2024. All participants provided written informed consent at enrollment.

### Participants

The number of participants we aimed to enroll was determined based on results from a prior open-label study of oxaloacetate for fatigue reduction (Cash and Kaufman, 2022). Assuming a standard deviation of 4 points (Cohen’s d = 0.625), a two-tailed alpha of 0.05, and 80% power to detect a between-group difference using a two-sample *t*-test, the required sample size was estimated to be 45 participants per group. Allowing for a 10% dropout rate, the final target enrollment was 50 participants per group. We successfully enrolled 69 male and female participants between 18 and 65 years of age. A total of 35 participants were randomized to the treatment arm (OAA group), and 34 participants were randomized to the control arm (control group). The participants were diagnosed with long COVID by a provider following suspected, probable, or confirmed infection with SARS-CoV-2, as defined by the WHO. The eligibility criteria included meeting the long COVID case definition as defined by the WHO (2022) (suspected, probable, or confirmed SARS-CoV-2 infection), experiencing moderate to severe fatigue and PEM, having access to a smartphone and internet, and willingness to comply with study procedures. The exclusion criteria were uncontrolled medical or psychiatric conditions, recent stimulant or oxaloacetate use, pregnancy or breastfeeding, significant recent head trauma, and a BMI of >40.

### Intervention

The investigational product was anhydrous enol-oxaloacetic acid, chemically identical to the oxaloacetate found in the body. The term oxaloacetate (OAA) is used to describe the investigational product. The participants were randomized to receive a daily oral dose of either 2,000 mg of OAA or 2,000 mg of white rice flour (control). OAA was administered as 500 mg capsules containing anhydrous enol-oxaloacetate, which undergoes chemical tautomerization to enol and keto forms in the acidic gastric environment (Bunik and Fernie, 2009). Both forms are naturally occurring metabolites that participate in cellular energy production via the citric acid cycle. The control capsules were the same shape and color as the OAA capsules and contained 500 mg of rice flour. The participants were instructed to take two 500 mg capsules with breakfast and two 500 mg capsules with lunch each day for the trial. The participants were provided with a 30-day supply of OAA or control capsules at each in-person visit. Compliance was monitored during in-person visits by collecting participant bottles and counting the remaining capsules to determine the number consumed. Any side effects from either OAA or the control were recorded. The were two withdrawals out of 35 participants in the OAA group and five out of 34 in the control group. During the study, 84% of the oxaloacetate group and 86% of the control group were compliant with dosing based on pill counts.

### Outcome measures

The primary outcome was a reduction in fatigue from baseline to the end of treatment, measured by the Chalder Fatigue Questionnaire (CFQ). The CFQ assesses physical and cognitive fatigue using an 11-item Likert scale, with a total score range of 0–33 (Chalder et al., 1993). The secondary outcomes included a reduction in symptom burden from baseline to the end of treatment, assessed with the RAND-36, which measures health-related quality of life across eight domains (e.g., energy, pain, and social functioning) (Hays and Morales, 2001), and the DePaul Symptom Questionnaire Short Form (DSQ-SF), which captures symptom burden across multiple domains relevant to long COVID (Oliveira et al., 2023). Total and fatigue domain scores were analyzed, and a responder definition (≥10% improvement) was used for *post hoc* analyses (Sunnquist et al., 2019). The exploratory outcomes included an assessment of cognitive performance using the Defense Automated Neurobehavioral Assessment (DANA) Brain Vital, an objective cognitive measure that includes simple reaction time (SRT), procedural reaction time (PRT), and go/no-go (GNG) tasks (Lathan et al., 2013), as well as physical function measured by upright activity (UP Time) using a wearable device (Palombo et al., 2020).

At Visits 1, 2, and 3, the participants completed the CFQ, RAND-36, and DSQ-SF assessments. The DANA Brain Vital assessment was also conducted at each in-person visit. Upon arrival at the BHC, the participants downloaded the DANA Brain Vital app to their smartphones (Lathan et al., 2013). The DANA Brain Vital is an FDA-cleared test that includes three measures of reaction time and information processing: simple reaction time (SRT), procedural reaction time (PRT), and sustained attention or GNG (Resnick and Lathan, 2016). Individual test results are reported as a cognitive efficiency score (calculated by accuracy × speed × 60,000) and a summary total cognitive efficiency score, which is the sum of the three cognitive efficiency tests. The SRT is a simple reaction time task in which the user taps an orange target symbol as soon as it appears on the screen. The PRT task incorporates choice by having the user differentiate between two sets of characters: when a 2, 3, 4, or 5 appears on the screen, the user taps one of two buttons—(2 or 3) or (4 or 5). The GNG task is a forced-choice measure of reaction time where either a gray foe or a green friend appears on the screen. The user is instructed to tap the screen only when the gray foe appears.

At the end of each in-person visit, the participants were given a fully charged wearable device, which was worn on the ankle to continuously measure UP Time for 7 days after the visit. The percentage of time the participants spent in an upright position (UP Time), where upright was defined as having their lower legs vertical with their feet on the floor, was measured over 24 h. The participants were asked to wear the device continuously on the outer side of their lower right ankle for 7 days. The wearable device was removed only during bathing or showering, during which the participants were instructed to position it as if standing while showering or as if lying down while bathing. At the end of the 7-day period, the participants returned the wearable device to the BHC via mail, where the raw data were processed and stored. A detailed description of the hardware, data collection, and data management system for UP Time has been previously published (Palombo et al., 2020; Sun et al., 2024).

### Statistical analysis

REDCap was the electronic data capture system for this trial (Harris et al., 2009). Statistical analyses were conducted using Python (v3.11) with the Pandas, Scipy, Statsmodels, and Seaborn packages. The data were summarized as means, standard deviations, standard errors, and 95% confidence intervals. Baseline demographic variables were compared between the treatment groups using chi-squared tests for categorical variables; *p*-values were reported to assess group equivalence. Within-group and between-group differences were examined across the time points, and effect sizes (Cohen’s d) were calculated to evaluate the magnitude of change. The outcome measures were analyzed using repeated measures ANOVA (RM-ANOVA) with Tukey’s *post hoc* HSD tests and linear mixed-effects models (LMMs) to evaluate the effects of time (Visit), treatment group (OAA vs. control), and their interaction. Although multivariate normality is a theoretical assumption of RM-ANOVA, our analysis involved univariate repeated measures across the three time points. RM-ANOVA is generally robust to moderate deviations from normality under these conditions, particularly with balanced group sizes and short timeframes (Blanca et al., 2017). Normality and homoscedasticity were assessed through visual inspection of residuals, and the robustness of the findings was verified by re-running models using LMMs.

Cognitive function was assessed using the DANA Brain Vital. To account for baseline variability, all scores were normalized to Visit 1 and expressed as a percent change from baseline. Between-group differences in the percent change were analyzed using independent samples *t*-tests, and the results were visualized as boxplots. Due to non-normal distribution and the presence of outliers in the DANA Brain Vital cognitive efficiency scores, median and interquartile range (IQR) were used to summarize central tendency and variability.

To explore the relationship between fatigue reduction and cognitive improvement, we conducted a responder analysis. The DSQ-SF responders were defined as participants achieving a ≥ 10% reduction in total symptom burden from Visit 1 to Visit 3. Linear regression models were used to evaluate whether the DSQ-SF responder status predicted percent change in the total cognitive efficiency score by Visit 3. Regression models were stratified by treatment group to assess whether this relationship differed between the OAA and control arms. Model coefficients (*β*₁) represent the mean difference in percent cognitive improvement between the responders and non-responders. R-squared values and *p*-values were reported to assess model fit and statistical significance. All statistical tests were two-tailed, with significance defined as a *p*-value < 0.05.

## Results

**Figure 1:**
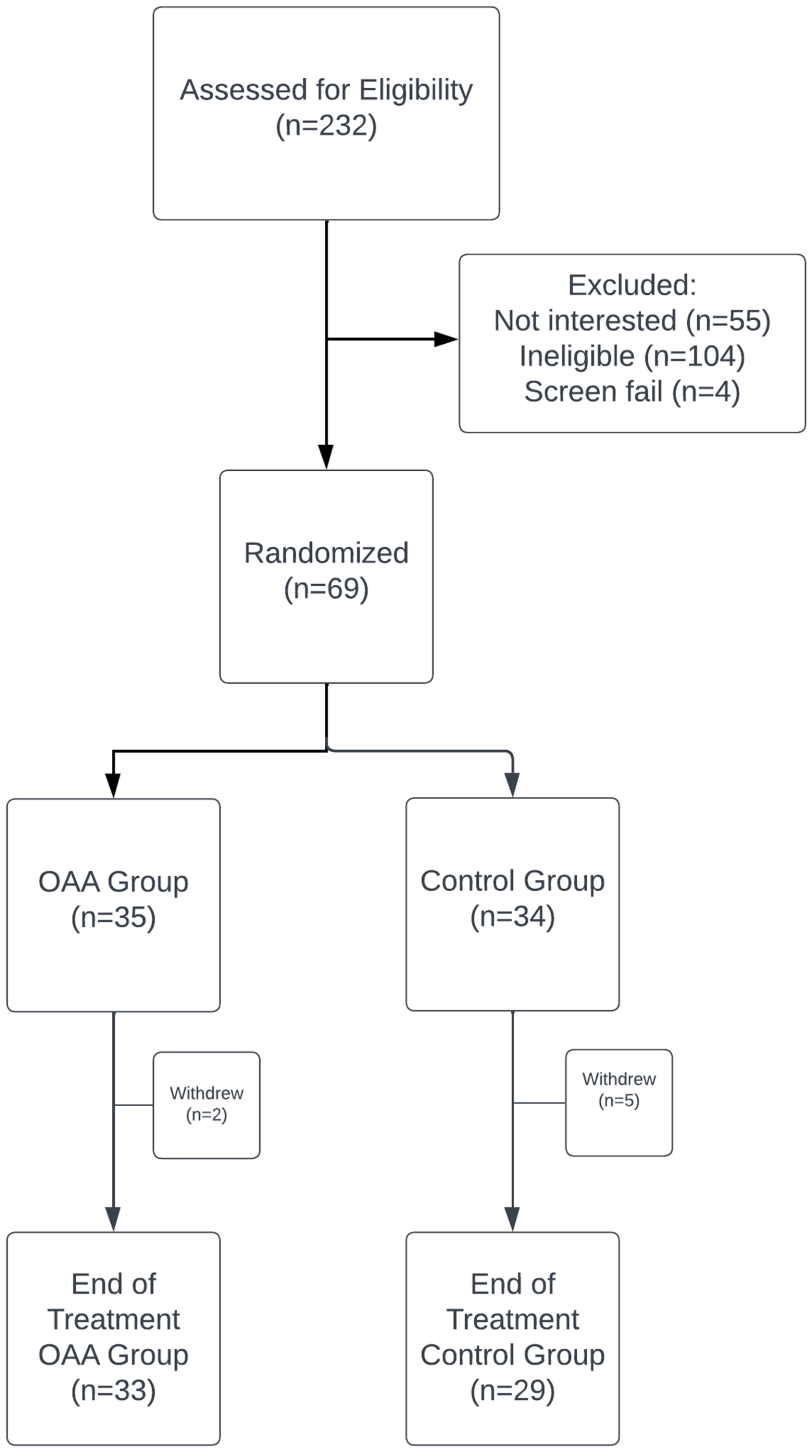
Participant flow through the clinical trial.

There were 69 participants enrolled in this trial, with 35 randomized to the OAA group and 34 randomized to the control group (Figure 1). Two participants in the OAA group were withdrawn early from the trial because of non-compliance and loss to follow-up. There were five participants in the control group who withdrew early for the following reasons: one participant did not want to participate after learning that OAA was chemically synthesized, one participant relocated and could not come to in-person visits, one participant experienced headaches, agitation, and facial numbness possibly related to trial participation, one participant experienced extreme exhaustion soon after taking the investigational product, and one participant was non-compliant, having stopped the investigational product without notifying the study staff. Baseline demographic characteristics were largely balanced between the OAA and control groups, with no statistically significant differences observed in sex, ethnicity, education, illness duration, or most employment categories. The only significant difference was a higher proportion of participants in the control group who were currently working (*p* = 0.038; Table 1).

**Table 1:** Participant demographics.

	OAA (*n* = 35)	Control (*n* = 34)	*P*-value
Sex			
Female	23 (66%)	26 (76%)	0.472
Male	12 (34%)	8 (24%)	0.472
Age			
Mean (std)	43 (14)	46 (12)	
Ethnicity			
White	32 (91%)	31 (91%)	1.000
Asian	1 (3%)	1 (3%)	1.000
Some other ethnicity	1 (3%)	2 (6%)	1.000
Unknown	1 (3%)	0 (0%)	1.000
Long COVID diagnosis			
Yes	34 (97%)	34 (100%)	1.000
No	1 (3%)	0 (0%)	1.000
Duration of illness			
3 to 6 months	2 (6%)	1 (3%)	1.000
6 months to 1 year	2 (6%)	1 (3%)	1.000
1 to 2 years	9 (26%)	5 (15%)	0.402
2 to 3 years	22 (63%)	27 (79%)	0.211
Marital status			
Married or living with partner	24 (68.5%)	19 (56%)	0.401
Never married	7 (20%)	10 (29%)	0.530
Divorced or separated	3 (8.5%)	3 (9%)	1.000
Widowed	0 (0%)	1 (3%)	0.988
Not reported	1 (3%)	1 (3%)	1.000
Education level			
High school graduate	2 (6%)	1 (3%)	1.000
Associate degree	6 (17%)	3 (9%)	0.504
College, no degree	6 (17%)	9 (26%)	0.517
College or professional degree	21 (60%)	21 (62%)	1.000
Employment			
Working	15 (43%)	24 (71%)	0.038
Unemployed	3 (8.5%)	1 (3%)	0.627
Disabled	5 (14%)	5 (15%)	1.000
Student	3 (8.5%)	1 (3%)	0.627
Other (e.g., keeping house, retired)	9 (26%)	3 (9%)	0.125

Fatigue, as measured by the CFQ, was the primary outcome. There were no statistically significant differences in improvement between the OAA and control groups for any individual CFQ item or for the CFQ total score (Table 2). The between-group effect size (Cohen’s d) for the change in the total CFQ score from Visit 1 to Visit 3 was −0.093, indicating a negligible and non-significant difference in fatigue reduction between the groups.

**Table 2:** Mean change in the CFQ item scores from visit 1 to visit 3 (negative mean change value = symptom improvement).

CFQ item	OAA (mean ± SE)	Control (mean ± SE)	t-statistic	*P*-value
Tired	−0.76 ± 0.16	−0.66 ± 0.16	−0.45	0.650
Rest	−0.79 ± 0.14	−0.59 ± 0.15	−0.92	0.360
Sleepy	−0.70 ± 0.15	−0.44 ± 0.18	−1.12	0.270
Starting	−0.48 ± 0.15	−0.66 ± 0.15	0.81	0.420
Energy	−0.73 ± 0.14	−0.75 ± 0.17	0.10	0.920
Strength	−0.67 ± 0.16	−0.66 ± 0.18	−0.04	0.970
Weak	−0.45 ± 0.17	−0.88 ± 0.15	1.84	0.070
Concentrate	−0.64 ± 0.16	−0.53 ± 0.17	−0.44	0.660
Slips	−0.58 ± 0.15	−0.25 ± 0.12	−1.70	0.100
Word find	−0.85 ± 0.17	−0.59 ± 0.13	−1.18	0.240
Memory	−0.73 ± 0.17	−0.63 ± 0.16	−0.44	0.660
Total Chalder score	−7.36 ± 1.40	−6.63 ± 1.38	−0.38	0.710

The RAND-36 was used as a secondary outcome measure to assess health-related quality of life. Of the eight domains evaluated, only the energy domain showed a statistically significant between-group difference. RM-ANOVA for the energy domain revealed a significant main effect of time (F(2,126) = 28.3, *p* < 0.001) but no significant main effect of group (F(1,63) = 2.9, *p* = 0.09) or group × time interaction (F(2,126) = 0.82, *p* = 0.44). The Tukey *post hoc* tests showed that the between-group difference in energy scores was statistically significant only at Visit 1, where the OAA group reported lower energy levels (mean ± SE: 10 ± 2.3) than the control group (18 ± 2.1; *p* = 0.01) (Table 3). No significant differences were observed at Visits 2 or 3, although mean energy scores remained numerically higher in the control group.

**Table 3:** RAND-36 domain scores by group and visit with the Tukey *post hoc* comparisons (mean ± SE).

Domain	Visit	OAA (mean ± SE)	Control (mean ± SE)	T-statistic	*P*-value
Physical function	1	41 ± 4.2	45 ± 3.9	−0.69	0.49
	2	46 ± 3.8	48 ± 4.1	−0.24	0.81
	3	46 ± 3.7	53 ± 4.2	−1.24	0.22
Role physical	1	1 ± 0.5	1 ± 0.5	−0.61	0.55
	2	7 ± 1.9	13 ± 2.1	−1.04	0.30
	3	10 ± 2.2	18 ± 2.4	−1.16	0.25
Energy	1	10 ± 2.3	18 ± 2.1	−2.66	**0.01**
	2	20 ± 2.0	27 ± 2.3	−1.52	0.13
	3	21 ± 2.1	30 ± 2.2	−1.62	0.11
Pain	1	47 ± 3.6	54 ± 3.8	−1.27	0.21
	2	52 ± 3.4	59 ± 3.7	−0.98	0.33
	3	53 ± 3.5	57 ± 3.9	−0.69	0.49
Emotion	1	50 ± 3.5	56 ± 3.4	−1.25	0.22
	2	58 ± 3.1	60 ± 3.3	−0.50	0.62
	3	58 ± 3.2	63 ± 3.6	−1.10	0.28
Role emotional	1	29 ± 2.8	27 ± 3.0	0.12	0.91
	2	29 ± 3.0	40 ± 3.1	−0.99	0.33
	3	38 ± 3.1	47 ± 3.4	−0.79	0.43
Social function	1	23 ± 3.1	31 ± 3.0	−1.71	0.09
	2	29 ± 2.9	39 ± 3.2	−1.52	0.13
	3	36 ± 3.0	40 ± 3.1	−0.57	0.57
General health	1	31 ± 2.7	29 ± 2.9	0.63	0.53
	2	31 ± 2.6	32 ± 2.7	−0.23	0.82
	3	33 ± 2.8	34 ± 2.8	−0.25	0.80

Bold values are statistically significant.

**Figure 2:**
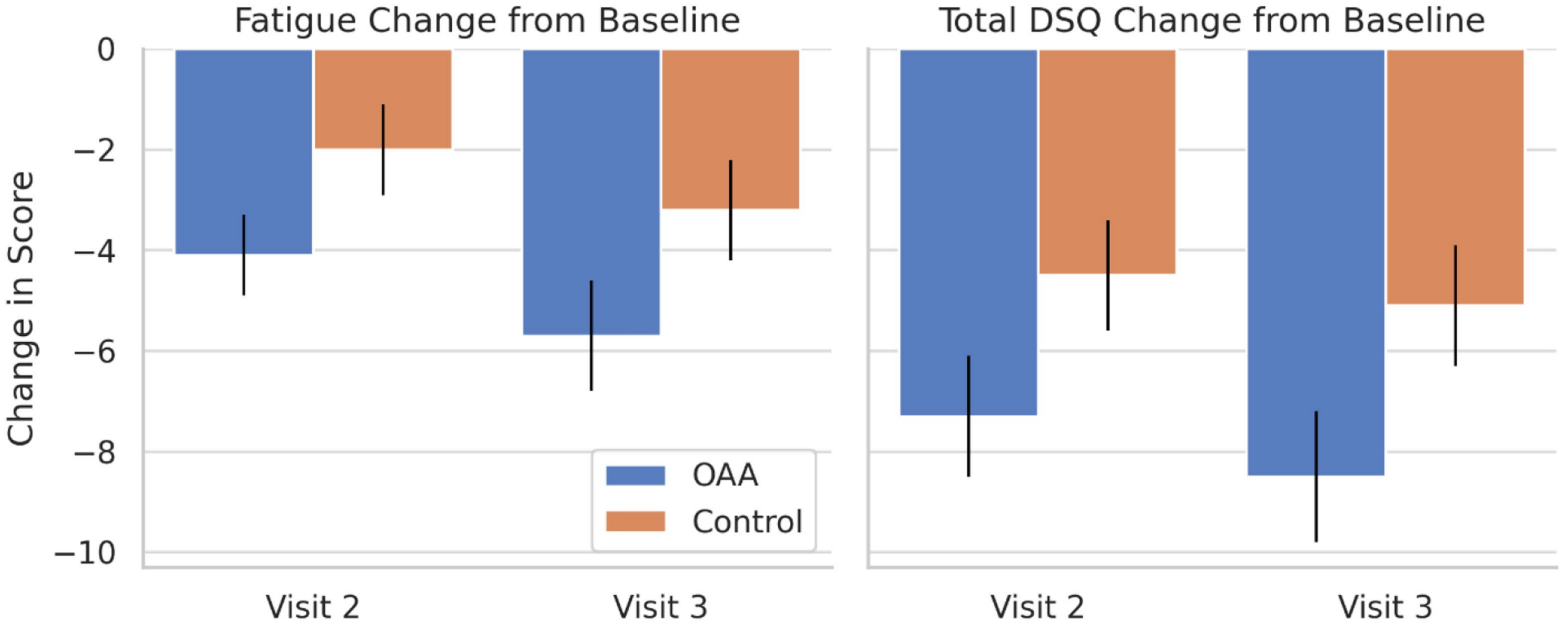
Change-from-baseline (delta) in the DSQ-SF fatigue and total symptom scores by visit and treatment group. Bar plots show the mean change from baseline (Visit 1) for the DSQ-SF fatigue and total symptom scores at Visits 2 and 3, comparing the OAA and control groups. Error bars represent standard errors. Repeated measures ANOVA revealed significant group × time interactions for both fatigue (*p* = 0.005) and total symptom burden (*p* = 0.028), indicating that the participants in the OAA group experienced earlier and greater symptom improvements. Tukey *post hoc* comparisons confirmed that the OAA group had significantly lower fatigue scores at Visit 2 (*p* = 0.012). The DSQ responder rates (defined as a ≥ 10% reduction in the total DSQ-SF score from Visit 1 to Visit 3) were 63% in the OAA group and 41% in the control group.

The DSQ-SF was used to assess fatigue and total symptom burden (Figure 2). RM-ANOVA revealed a significant main effect of time for both fatigue (F(2,122) = 22.4, *p* < 0.001) and total symptom burden (F(2,122) = 18.7, *p* < 0.001), as well as significant group × time interactions (fatigue: F(2,122) = 5.6, *p* = 0.005; total DSQ-SF score: F(2,122) = 3.7, *p* = 0.028), indicating that the OAA group experienced greater symptom improvements over time compared to the control group. The main effect of group was not statistically significant (fatigue: *p* = 0.119; total DSQ-SF score: *p* = 0.143), suggesting similar average symptom levels across the groups. The Tukey *post hoc* comparisons confirmed that fatigue scores were significantly lower in the OAA group compared to the control group at Visit 2 (*p* = 0.012), indicating earlier symptom improvement in the treatment arm. By Visit 3, both groups had improved, and the between-group difference was no longer statistically significant (*p* = 0.088), although the OAA group continued to show numerically lower fatigue scores. Figure 2 displays the changes in fatigue and the total DSQ-SF symptom scores for each group at Visits 2 and 3. A responder analysis (defined as a ≥ 10% reduction in the total DSQ-SF score from Visit 1 to Visit 3) showed that 63% of the participants in the OAA group and 41% in the control group met the responder criteria; however, this difference was not statistically significant (*p* = 0.118).

UP Time was used as an exploratory outcome to assess the effects of oxaloacetate on time spent upright with one’s feet on the floor (including sitting with one’s feet on the floor). There was no statistically significant difference in average UP Time between the OAA and control groups at any visit.

**Figure 3:**
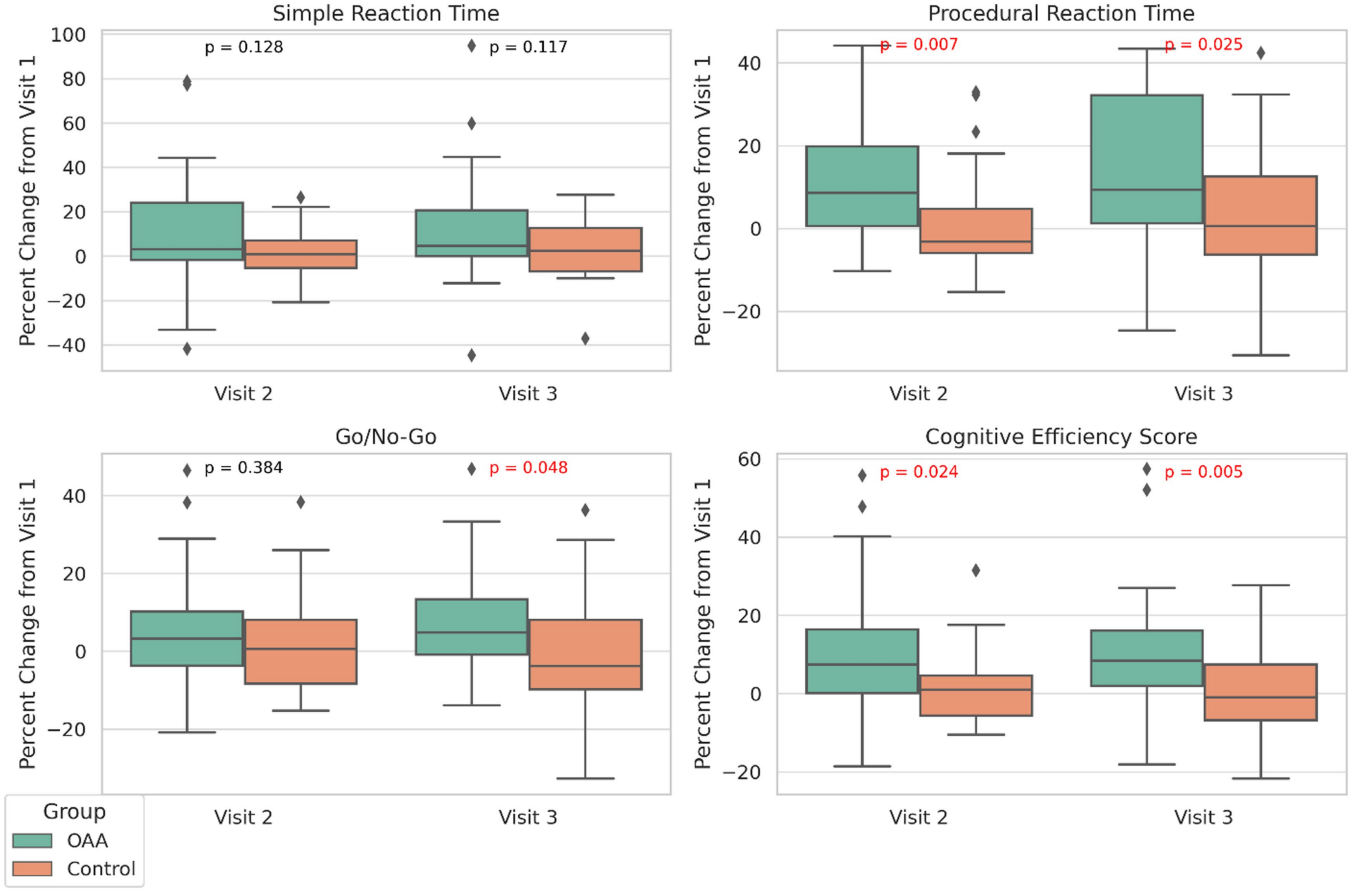
Percent change in cognitive performance measures from Visit 1 to Visits 2 and 3 for the OAA and control groups. Boxplots display the percent change for simple reaction time, procedural reaction time, go/no-go, and total cognitive efficiency scores. The changes are normalized to each participant’s Visit 1 score. Horizontal lines within boxes indicate medians; the boxes span the interquartile range, and whiskers extend to 1.5 × IQR. Outliers are plotted as individual points. Between-group comparisons were conducted using independent *t*-tests for each visit. Exact *p*-values are annotated within each panel. Red font indicates statistically significant differences (*p* < 0.05).

Brain fog, a common and debilitating symptom of long COVID, was assessed using the DANA Brain Vital. RM-ANOVA revealed significant main effects of time for all cognitive domains: SRT (F(2, 136) = 52.20, *p* < 0.0001), PRT (F(2, 136) = 38.62, *p* < 0.0001), GNG (F(2, 136) = 59.02, *p* < 0.0001), and total cognitive efficiency (F(2, 136) = 59.41, *p* < 0.0001), indicating overall improvement in cognitive performance across visits. To evaluate whether the OAA and control groups improved differently over time, an LMM including a group x visit interaction term was conducted. The percent changes in SRT, PRT, GNG, and Total Cognitive Efficiency scores are shown in Figure 3. SRT improved in both groups, and there were no between-group differences at either time point (*p* = 0.054 at Visit 2; *p* = 0.091 at Visit 3). The OAA group showed significantly greater improvement in PRT at Visit 2 (+10.5% vs. –0.3%; *p* = 0.012) and Visit 3 (+14.1% vs. –1.4%; *p* = 0.011) compared to the control group. There were no differences in GNG at Visit 2 (*p* = 0.547); however, by Visit 3, the OAA group demonstrated significantly greater improvement in GNG compared to the control group (+10.0% vs. +0.9%; *p* = 0.017). The between-group comparisons of the percent change in total cognitive efficiency revealed significantly greater improvement in the OAA group at both Visit 2 (mean change: +8.7% vs. +0.2%; *p* = 0.021) and Visit 3 (+10.7% vs. –0.04%; *p* = 0.007), indicating a robust cognitive benefit relative to the control group.

**Figure 4:**
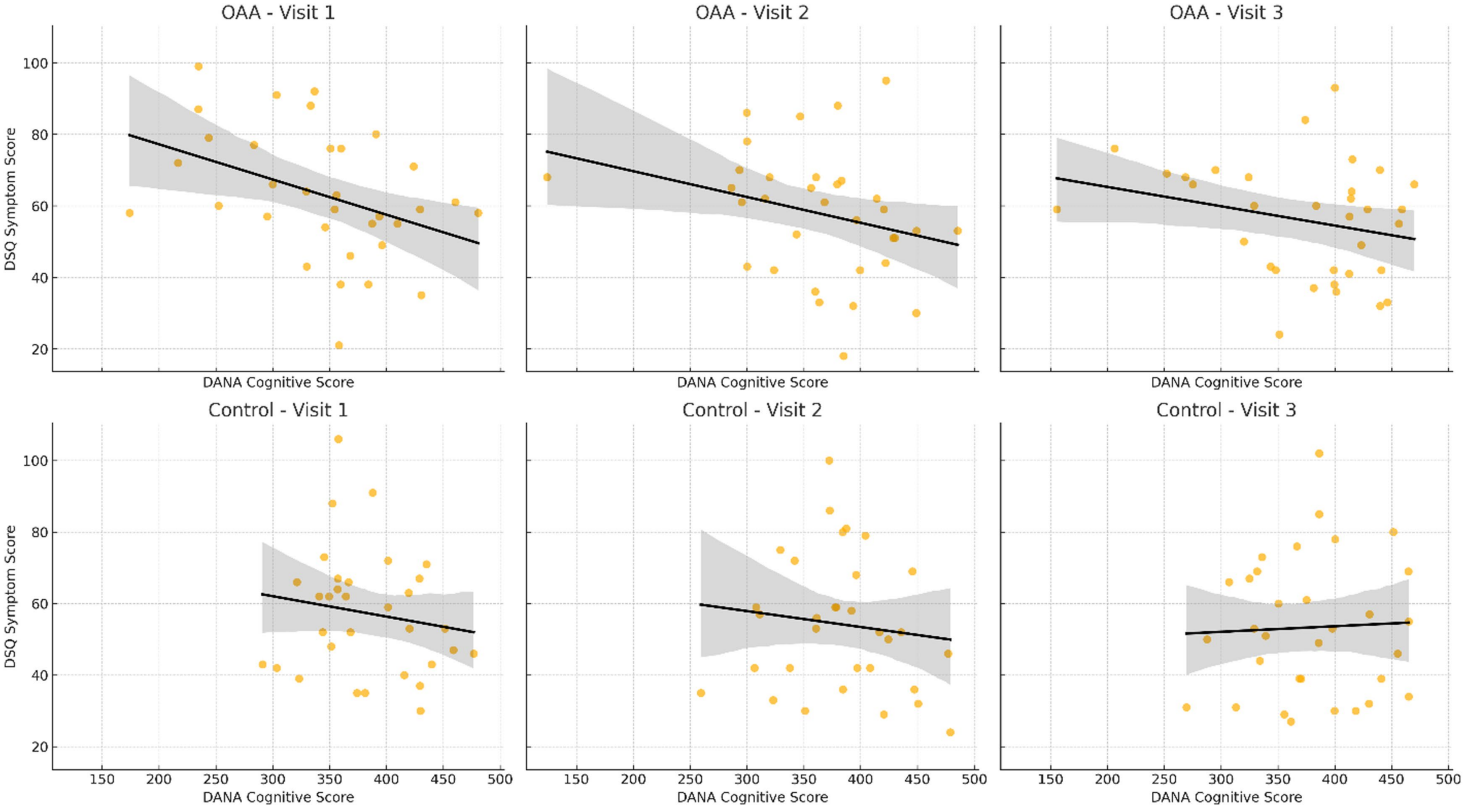
Correlation between the DANA cognitive scores and DSQ-SF symptom scores by treatment group and visit. Scatterplots with linear regression lines (black) illustrate the relationship between cognitive function (DANA brain vital scores) and symptom burden (total DSQ-SF scores) across the three study visits (Visits 1, 2, and 3) in the OAA and control groups. Each point represents an individual participant at a specific visit. In the OAA group, a statistically significant negative correlation was observed at Visit 1 (r = −0.40, *p* = 0.023), indicating that higher cognitive scores were associated with lower symptom severity. At Visits 2 and 3, the negative association persisted but was weaker and not statistically significant. In the control group, correlations were consistently weak and non-significant across all time points. These results suggest a stronger cognitive–symptom relationship in the OAA group, particularly at baseline.

To explore the relationship between cognitive function and symptom burden, Pearson correlation coefficients were calculated between the DANA Brain Vital cognitive scores and DSQ-SF symptom scores at each visit, stratified by treatment group (Figure 4). In the OAA group, a moderate and statistically significant negative correlation was observed at Visit 1 (r = −0.40, *p* = 0.023), indicating that the participants with greater symptom severity reported lower cognitive performance at baseline. This relationship weakened and was no longer significant at Visit 2 (r = −0.27, *p* = 0.123) or Visit 3 (r = −0.25, *p* = 0.153). In contrast, the control group exhibited weak and non-significant correlations across all time points, with r-values ranging from −0.12 to −0.15 (all *p* > 0.39). The Tukey *post hoc* comparisons confirmed that fatigue scores were significantly lower in the OAA group compared to the control group at Visit 2 (*p* = 0.012), indicating earlier symptom improvement in the treatment arm. By Visit 3, both groups had improved, and the between-group difference was no longer statistically significant (*p* = 0.088), although the OAA group continued to show numerically lower fatigue scores.

Among the OAA group, the DSQ-SF responder status was significantly associated with cognitive improvement. The DSQ-SF responders in the OAA group exhibited an average 19.8% greater increase in DANA cognitive scores at Visit 3 compared to the non-responders (*β* = 19.81, *p* = 0.0013), with an R^2^ of 0.29, indicating that nearly 29% of the variance in cognitive improvement was explained by symptom response. In contrast, among the control group, the association between the DSQ-SF response and DANA improvement was weaker and not statistically significant (β = 8.70, *p* = 0.1091, R^2^ = 0.08). These results suggest that in the context of the OAA treatment, reductions in symptom burden were more strongly associated with improvements in cognitive function.

Overall, treatment with OAA was well tolerated. Treatment-emergent adverse events (TEAEs) were experienced by 15 participants (43%) in the OAA group and 10 participants (29%) in the control group (Table 4). There was one SAE in the OAA group, costochondritis, which was reported as possibly related at Visit 2 and resolved by Visit 3. The majority of TEAEs were either mild (25%) or moderate (10%) in severity. TEAEs considered possibly related to the investigational product were reported for 10 (14%) of the total participants. No TEAEs were reported as probably related. The TEAEs experienced by the participants are listed in Table 5. Infections were the most common TEAEs among all participants, with five (7%) experiencing upper respiratory infections, three (4%) diagnosed with COVID-19, and two (3%) reporting cold symptoms. Gastrointestinal issues were the next most common TEAEs, with three (4%) participants experiencing abdominal pain, two (4%) experiencing vomiting, and one (1%) experiencing diarrhea. All other reported TEAEs occurred in only one participant each.

**Table 4:** Overview of treatment-emergent adverse events.

Category	OAA group (*n* = 35)	Control group (*n* = 34)	Total (*n* = 69)
Participants with at least one TEAE^a^	15 (43%)	10 (29%)	25 (36%)
Participants with at least one TEAE by severity^b^			
Mild	9 (26%)	8 (24%)	17 (25%)
Moderate	5 (14%)	2 (6%)	7 (10%)
Severe	1 (3%)	0 (0%)	1 (1%)
Participants with at least one TEAE by relationship^c^			
Not related	9 (26%)	7 (21%)	22 (32%)
Possibly related	4 (11%)	3 (9%)	10 (14%)
Probably related	0 (0%)	0 (0%)	0 (0%)
Participants with at least one SAE			
Not related	1 (3%)	0 (0%)	1 (1%)
Possibly related	0 (0%)	0 (0%)	0 (0%)
Probably related	0 (0%)	0 (0%)	0 (0%)
Participants who died	0 (0%)	0 (0%)	0 (0%)

a TEAEs are adverse events with a start date on or after the first dose of the study investigational product.

b Participants were counted only once at the worst severity.

c Participants were counted only once at the strongest relationship with the investigational product.

**Table 5:** Treatment-emergent adverse events reported for participants.

System organ class (preferred term)	OAA group (*n* = 35)	Control group (*n* = 34)	Total (*n* = 69)
Participants with at least one TEAE^a^			
Gastrointestinal			
Diarrhea	0 (0%)	1 (3%)	1 (1%)
Abdominal pain	2 (6%)	1 (3%)	3 (4%)
Vomiting	2 (6%)	0 (0%)	2 (3%)
Infections			
COVID-19	2 (6%)	1 (3%)	3 (4%)
Cold symptoms	1 (3%)	1 (3%)	2 (3%)
Upper respiratory infection	3 (9%)	2 (6%)	5 (7%)
Nervous system			
Headache	0 (0%)	1 (3%)	1 (1%)
Numbness on the left side of the head	0 (0%)	1 (3%)	1 (1%)
Motor tic	0 (0%)	1 (3%)	1 (1%)
Musculoskeletal			
Hip fracture	0 (0%)	1 (3%)	1 (1%)
Bilateral knee pain	1 (3%)	0 (0%)	1 (1%)
Costochondritis	1 (3%)	0 (0%)	1 (1%)
Muscle aches	1 (3%)	0 (0%)	1 (1%)
Thumb burning	1 (3%)	0 (0%)	1 (1%)
Skin			
Basal cell removal on the ankle	1 (3%)	0 (0%)	1 (1%)
Erythema annulare centrifugum	1 (3%)	0 (0%)	1 (1%)
General			
Fatigue	1 (3%)	1 (3%)	1 (1%)
Lightheadedness	1 (3%)	0 (0%)	1 (1%)
Chills	1 (3%)	0 (0%)	1 (1%)
Mast cell activation syndrome	0 (0%)	1 (3%)	1 (1%)

a TEAEs are adverse events with a start date on or after the first dose of the study investigational product.

## Discussion

This randomized, controlled trial evaluated the effects of OAA supplementation on fatigue, cognitive function, and symptom burden in individuals with long COVID. Although the primary outcome, fatigue reduction measured by the CFQ, did not reach statistical significance, the pattern of the results across the secondary and exploratory measures suggests potential clinical benefits of OAA for this population.

The CFQ showed similar improvements across both treatment arms, with a negligible between-group effect size. This underscores the limitations of the CFQ’s sensitivity in detecting treatment effects in long COVID, particularly given the complex, multidimensional nature of fatigue in this condition (Gladwell et al., 2024). In contrast, the DSQ-SF, a multidomain symptom instrument developed for ME/CFS and used to evaluate long COVID, detected significantly greater reductions in fatigue and total symptom burden in the OAA group by Visit 2 (McGarrigle et al., 2024). Although the between-group responder rate did not reach statistical significance, these findings underscore the importance of selecting outcome measures, such as the DSQ-SF, that are designed to detect clinically meaningful change in heterogeneous disease populations.

Additional insights were provided by the RAND-36, which measures health-related quality of life. Of the eight domains assessed, only the energy domain showed a statistically significant difference between the groups at baseline, with the OAA group reporting lower scores. However, this difference was not sustained over time, and no group × time interactions emerged, suggesting that the overall quality-of-life trajectories were similar across the groups. These findings reinforce the need for outcome measures that are sensitive to short-term symptom changes and tailored to the clinical features of long COVID.

Cognitive dysfunction, or “brain fog,” is a hallmark and highly disabling feature of long COVID (Thaweethai et al., 2023; Davis et al., 2021). At least 57% of individuals with long COVID reported experiencing cognitive symptoms daily and may continue to experience chronic cognitive symptoms for months or years (Zhao et al., 2024; Jaywant et al., 2024). The objective cognitive outcomes from this study provide further evidence of OAA’s potential therapeutic benefit. The participants who received OAA demonstrated significantly greater improvements in procedural reaction time, Go/No-Go performance, and total cognitive efficiency compared to the control group. These changes exceeded thresholds previously associated with functional cognitive recovery and emerged by Visit 2, suggesting a relatively rapid treatment response (Jaywant et al., 2024; Vidoni et al., 2021). Furthermore, cognitive improvements in the OAA group were significantly associated with reductions in symptom burden—an association not observed in the control group. These findings are consistent with prior evidence of the neuroprotective and neurometabolic benefits of oxaloacetate, such as its ability to stimulate mitochondrial biogenesis, reduce neuroinflammation, and increase neurological glucose uptake (Wilkins et al., 2014; Wilkins et al., 2016; Onuki et al., 2025).

Notably, a significant baseline correlation between cognitive performance and symptom severity was observed only in the OAA group, suggesting that symptom–cognition coupling may be particularly prominent in individuals with greater baseline impairment. This relationship attenuated over time, potentially reflecting a therapeutic decoupling effect, where improvements in systemic symptoms and cognitive dysfunction occur in parallel but independently, possibly due to OAA’s neurometabolic effects (Wilkins et al., 2016).

Despite improvements in symptom burden and cognitive function, UP Time, an objective proxy for time spent in an upright position, did not differ between the groups at any time point. UP Time has been shown to differentiate patients with ME/CFS based on disease severity and self-reported hours of upright activity (Palombo et al., 2020). Therefore, this may suggest that OAA’s effects are domain-specific, influencing cognition and subjective symptoms without translating into measurable changes in time spent upright.

OAA was well tolerated, with a safety profile comparable to the control. No serious adverse events were attributed to the intervention, and the majority of treatment-emergent events were mild to moderate.

This study has several limitations. The modest sample size increased the risk of baseline imbalances, including differences in employment status that may reflect varying levels of functional impairment. The 42-day duration may have been too short to capture changes in upright activity, physical function, or sustained symptom relief. In addition, the CFQ may have lacked sensitivity to detect meaningful treatment effects in this population. Finally, the single-site design and absence of biological markers limit the generalizability of the findings and mechanistic interpretation.

Overall, while the primary outcome was not met, the convergence of the DSQ-SF findings, objective cognitive improvements, and symptom–cognition coupling patterns provides encouraging evidence of OAA’s therapeutic activity. These findings support further investigation of OAA in larger, longer-duration trials using multidimensional and responsive outcome measures tailored to the complexity of long COVID.

## Conclusion

In conclusion, although the primary outcome of fatigue reduction measured by the CFQ did not reach statistical significance, the analyses of the secondary and exploratory outcomes provide evidence that OAA may offer clinically meaningful benefits for individuals with long COVID. These findings support the potential of OAA to improve symptom burden and cognitive function over a 42-day treatment period relative to control. The association between symptom response and cognitive improvement in the treatment group further supports the potential biological activity of OAA. These findings underscore the importance of including both subjective and objective endpoints, along with responder analyses and effect size estimates, to fully evaluate treatment effects in long COVID. Oxaloacetate was well tolerated, and the results from this trial support the need for larger, longer-duration studies to confirm its efficacy and further elucidate its mechanisms of action.

## Data Availability

The raw data supporting the conclusions of this article will be made available by the authors, without undue reservation.
